# Supplementary figures and images for: Data-driven analysis of fine-scale badger movement in the UK
Source: PLoS Comput Biol. 2025 Aug 28;21(8):e1013372. doi: 10.1371/journal.pcbi.1013372 (PMC12393748; doi:10.1371/journal.pcbi.1013372)

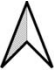

● Removed Data  
● Data

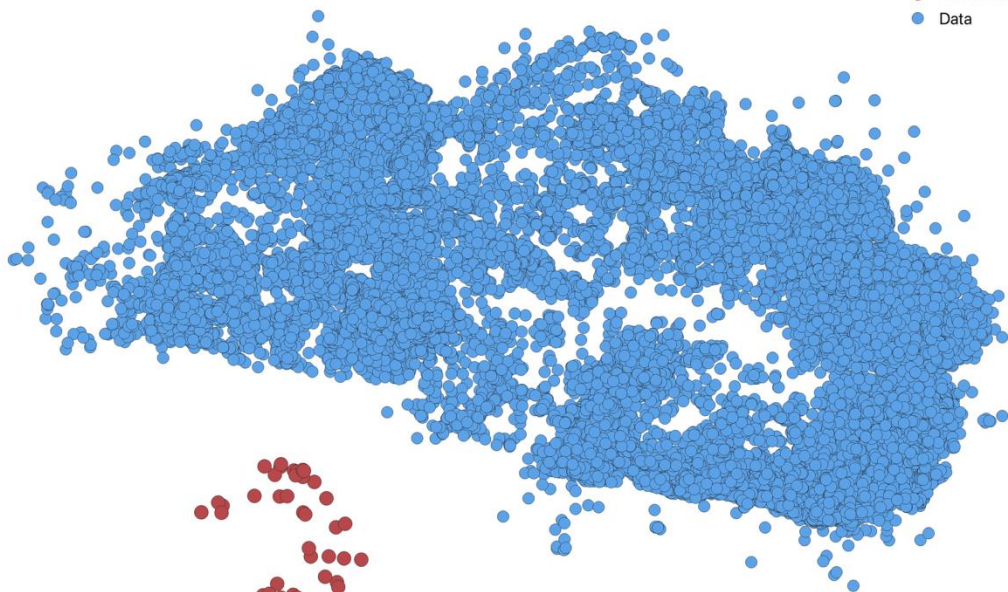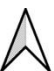

0 500 1,000 m

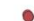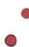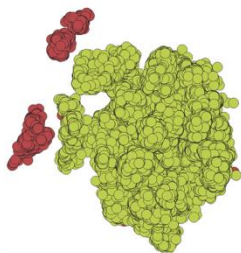

0 5 10 km

● Removed Data  
● Data

Supplement: S1 Fig — Each dot represents a specific date-time location for an individual badger. (PDF) [file pcbi.1013372.s001.pdf]

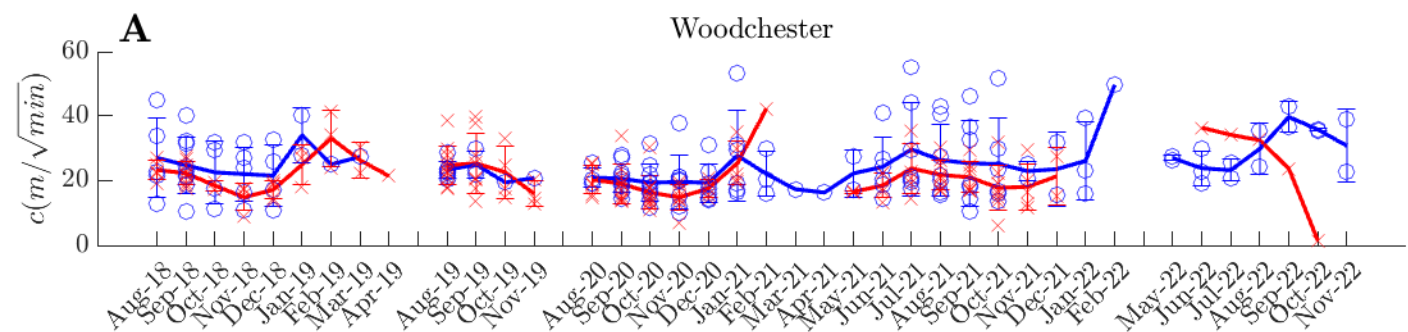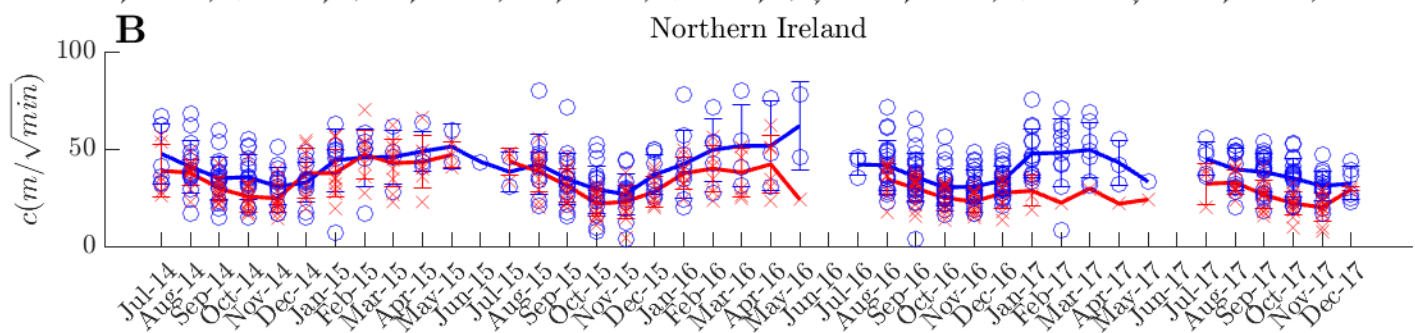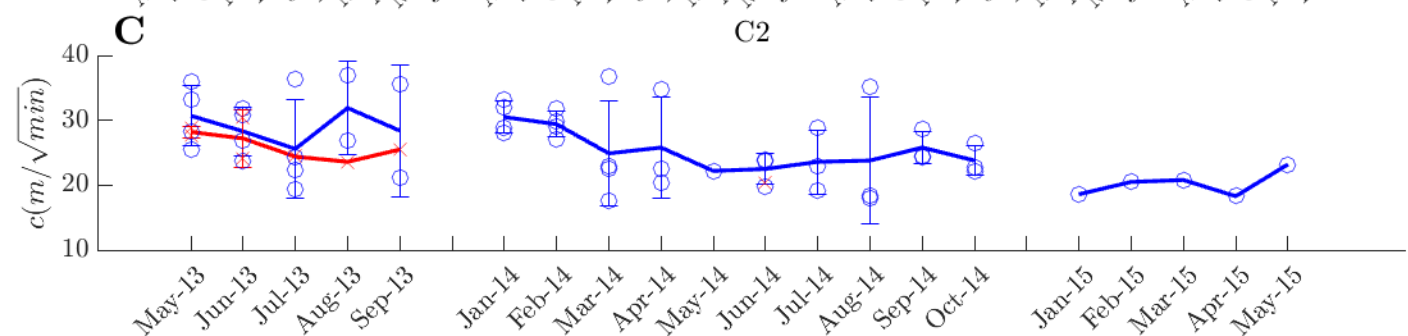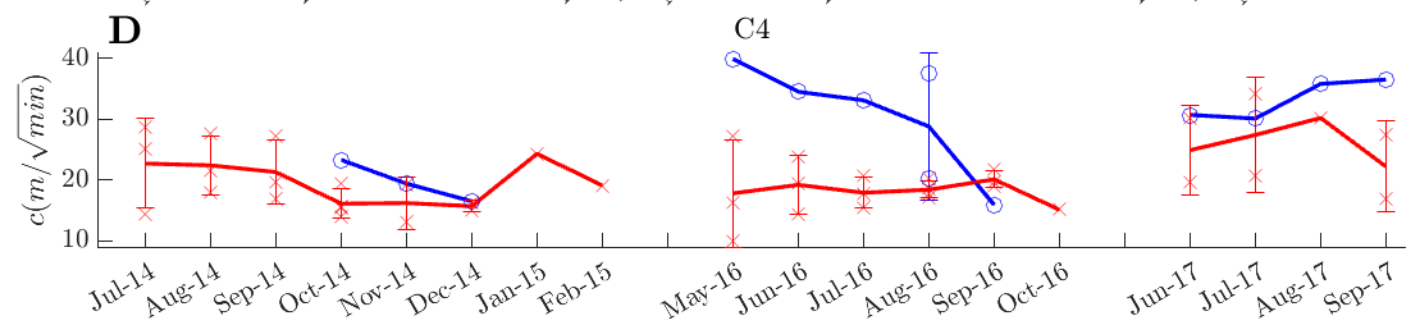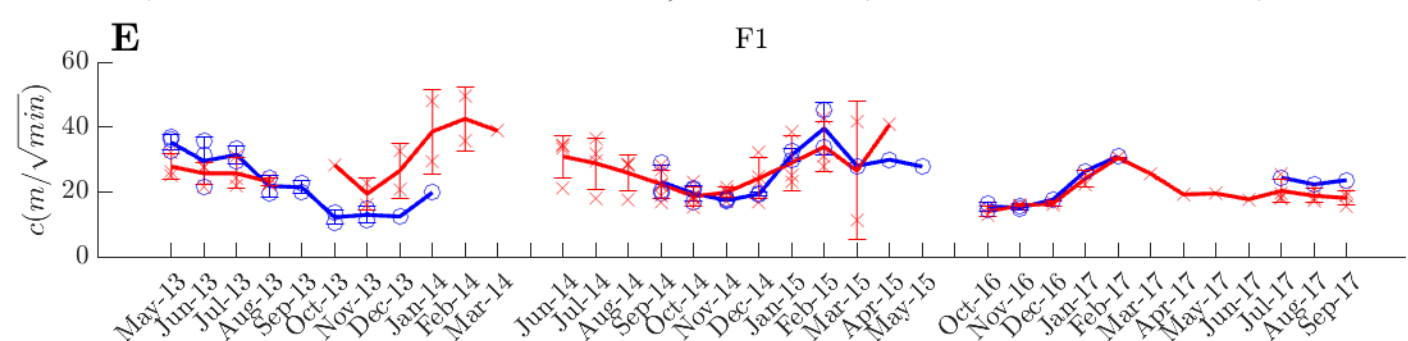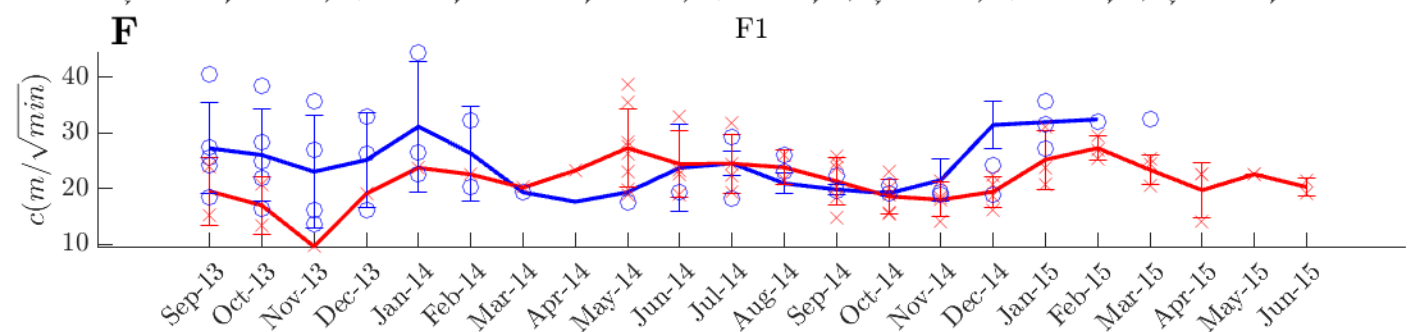

Supplement: S2 Fig — Individual diffusion values are shown by month and sex (circle for male and cross for female). Note, that the low estimation for the diffusion in Woodchester October 2022 comes from 12 data points originating from a single female badger. See Fig 1 and S3 Fig for report of number of fixes and count of badgers per month, respectively. (PDF) [file pcbi.1013372.s002.pdf]

# Woodchester

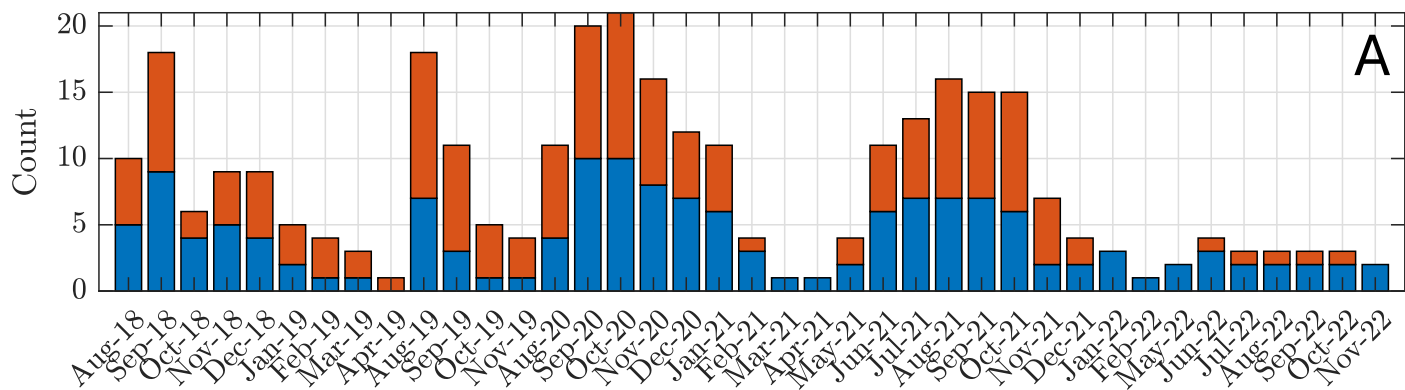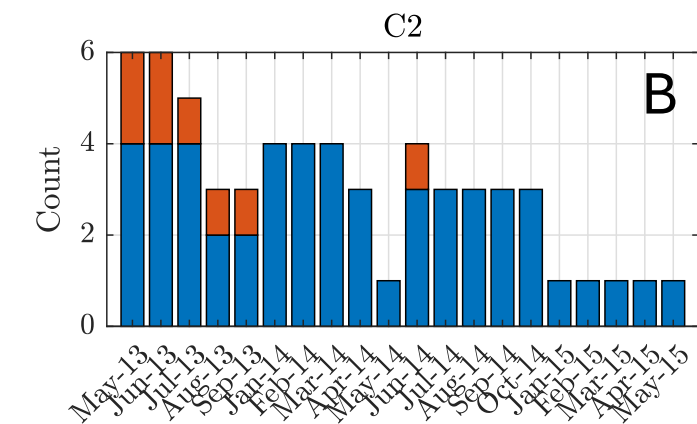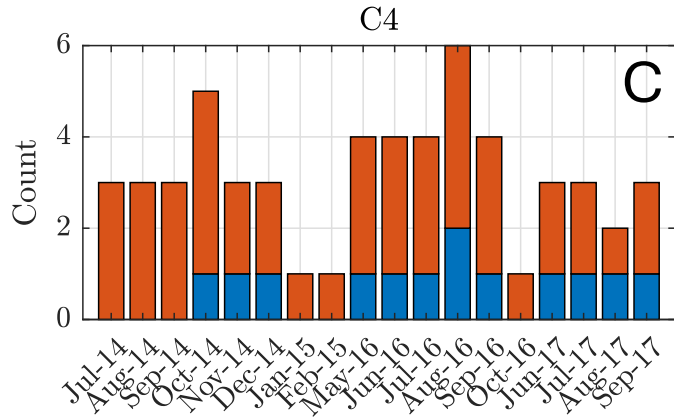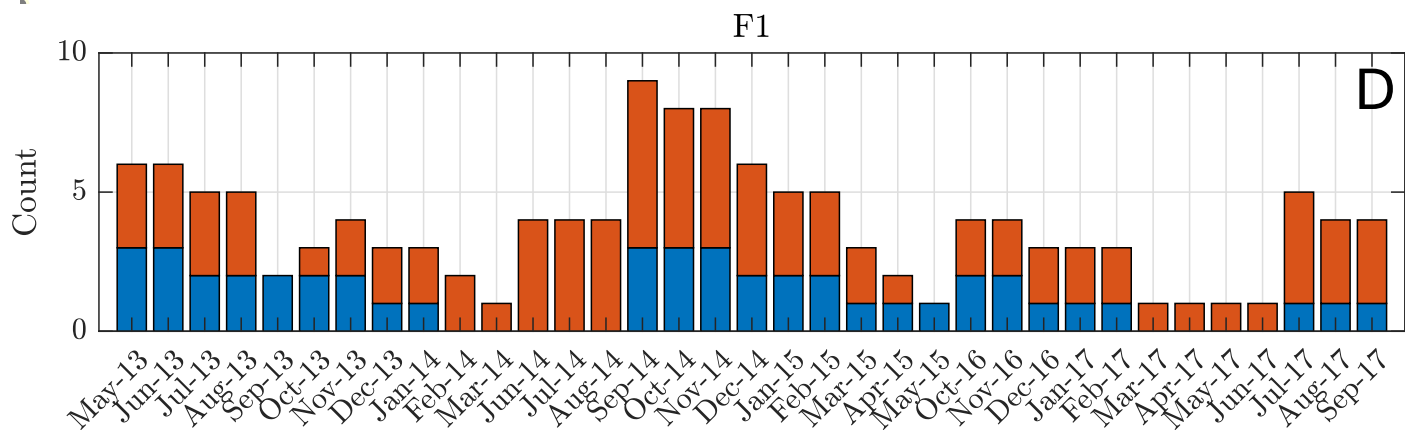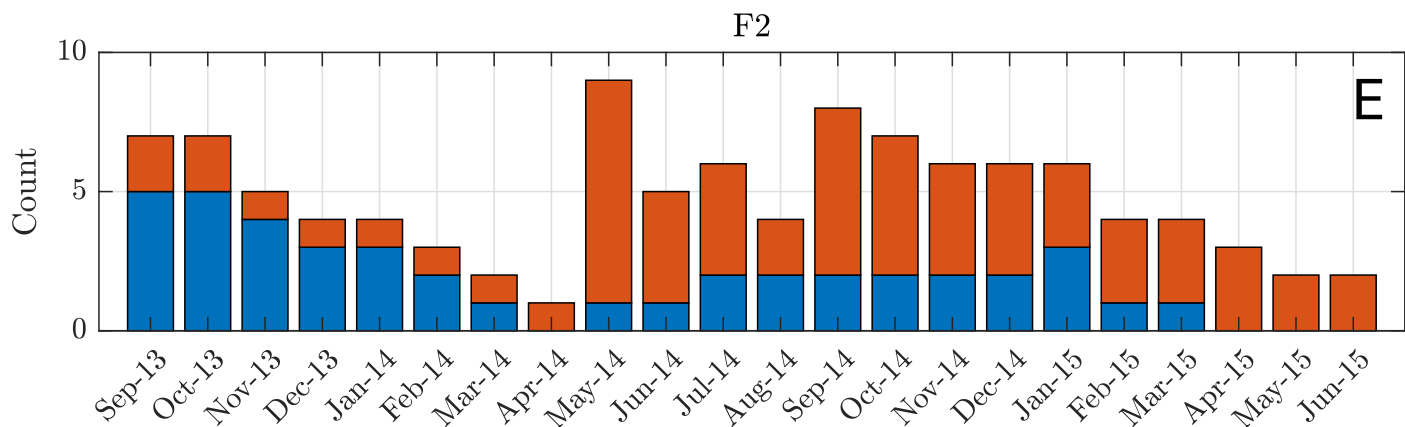

# Northern Ireland

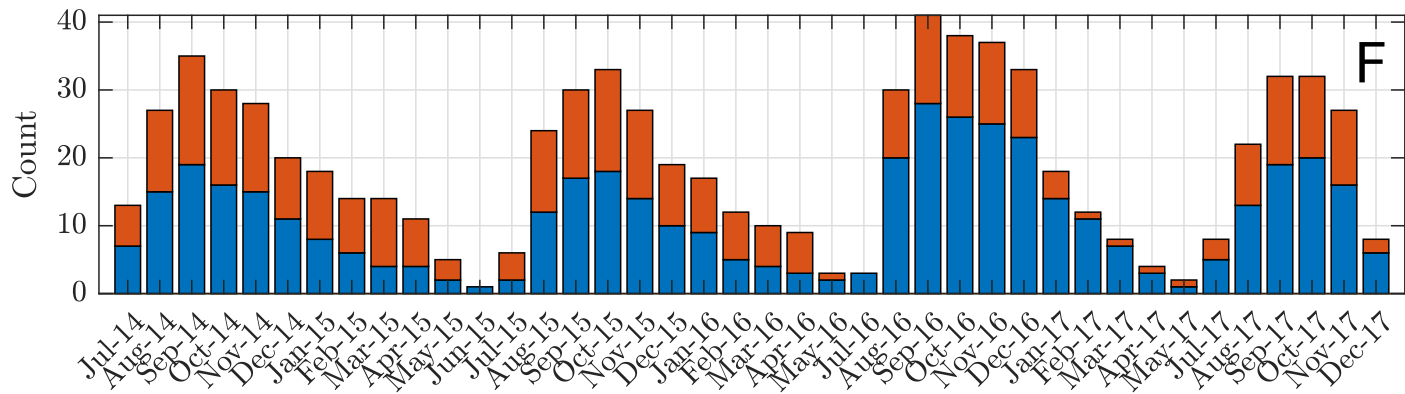

Supplement: S3 Fig — (PDF) [file pcbi.1013372.s003.pdf]

Plot of residuals vs. fitted values

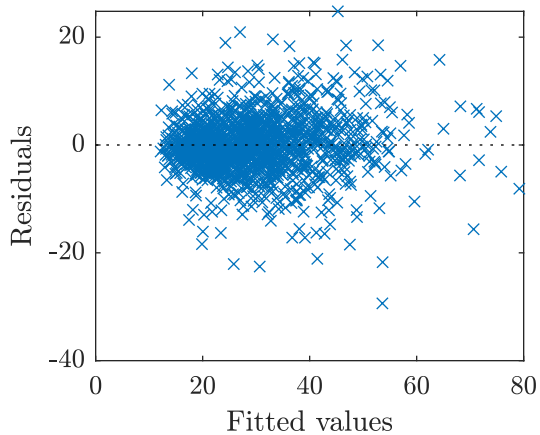

Histogram of residuals

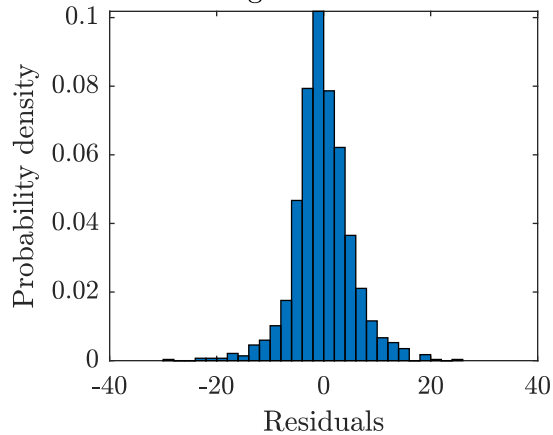

Residuals by Month

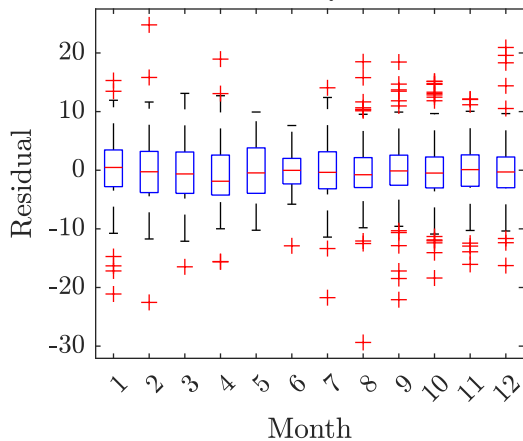

Residuals by Sex

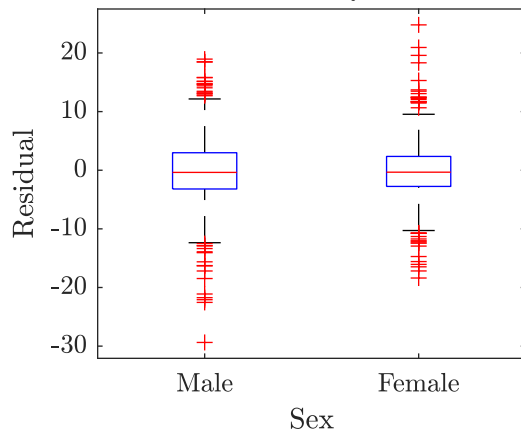

Supplement: S4 Fig — (PDF) [file pcbi.1013372.s004.pdf]

A

# Northern Ireland

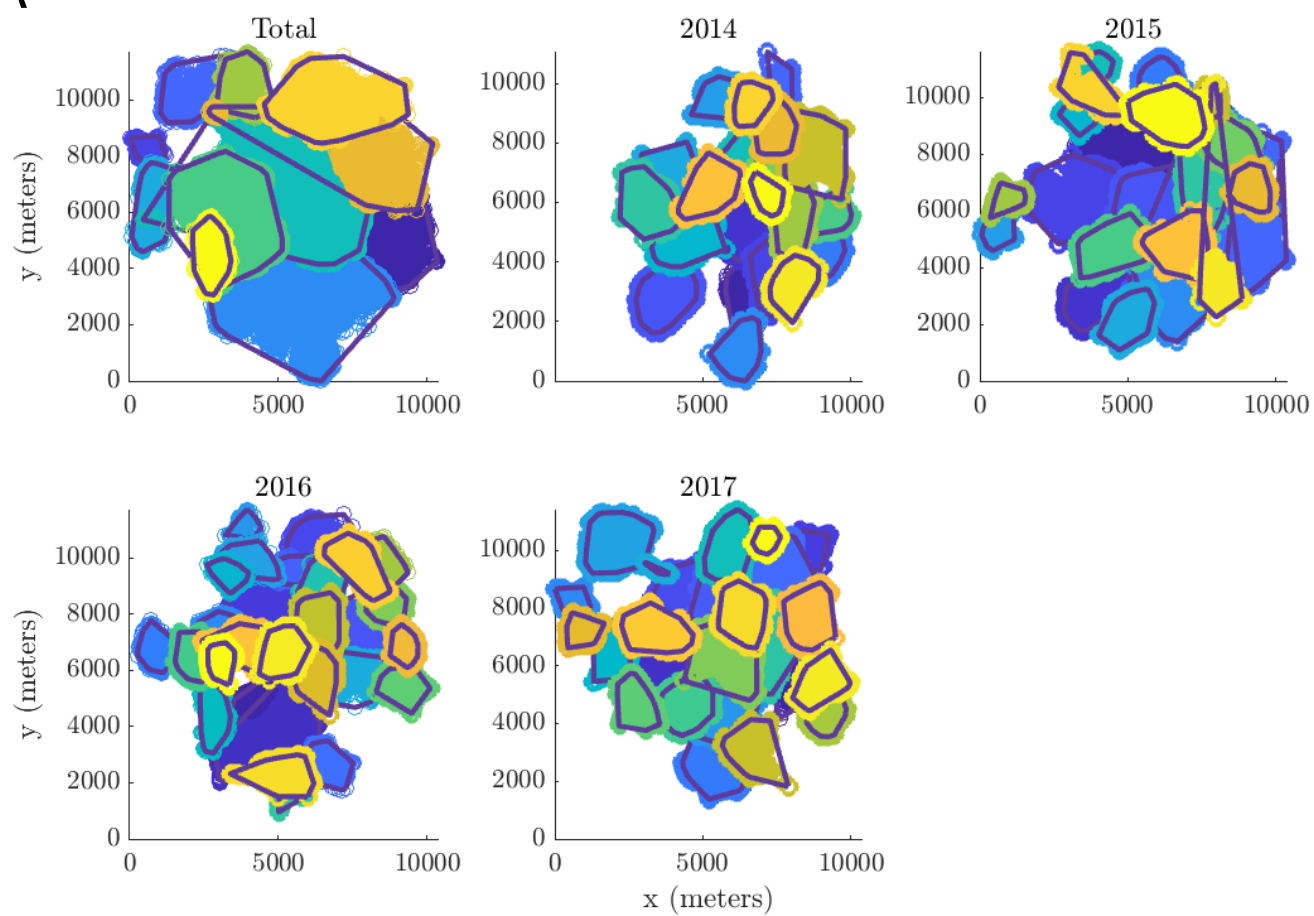

**B**

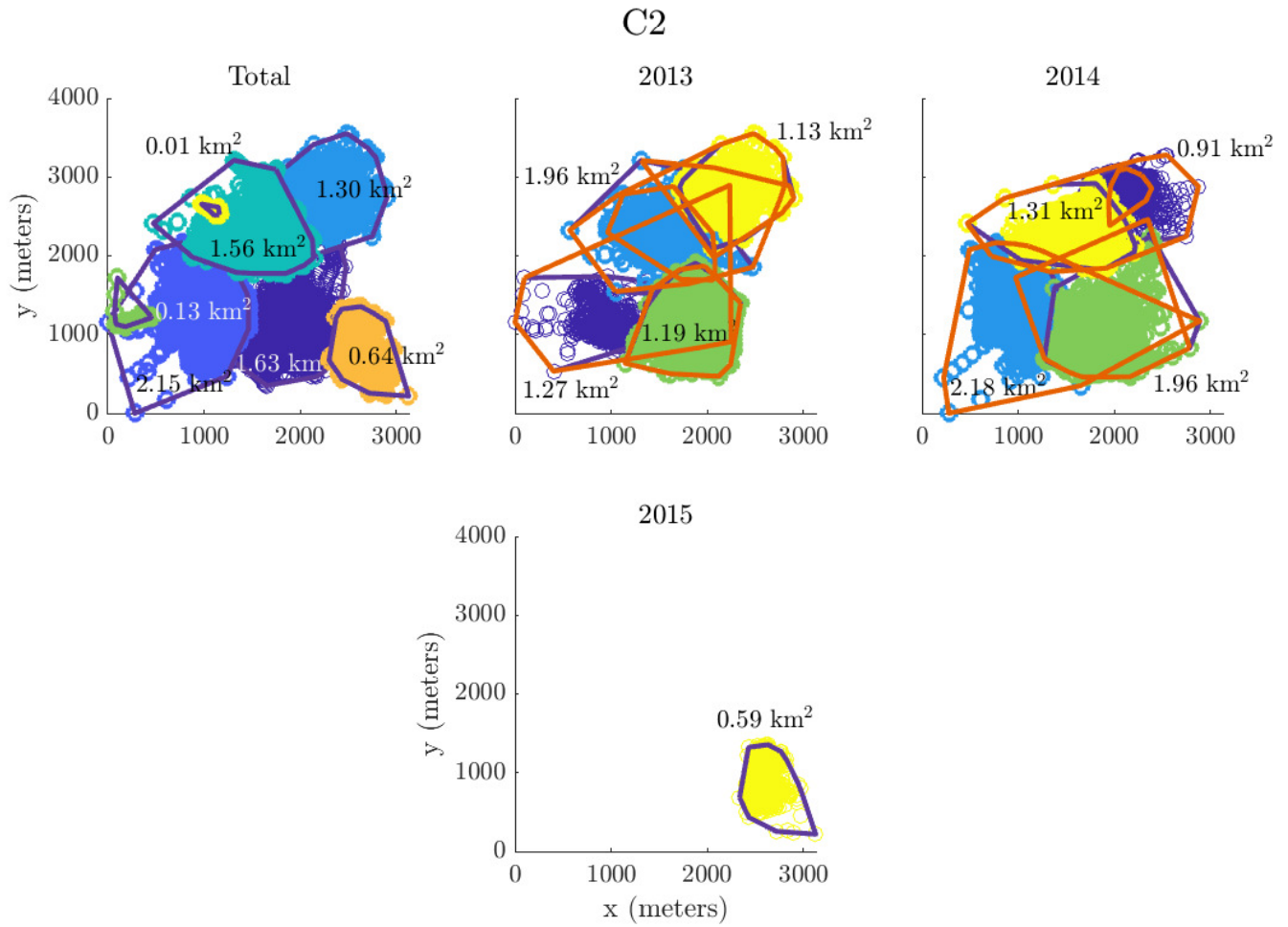

**C**

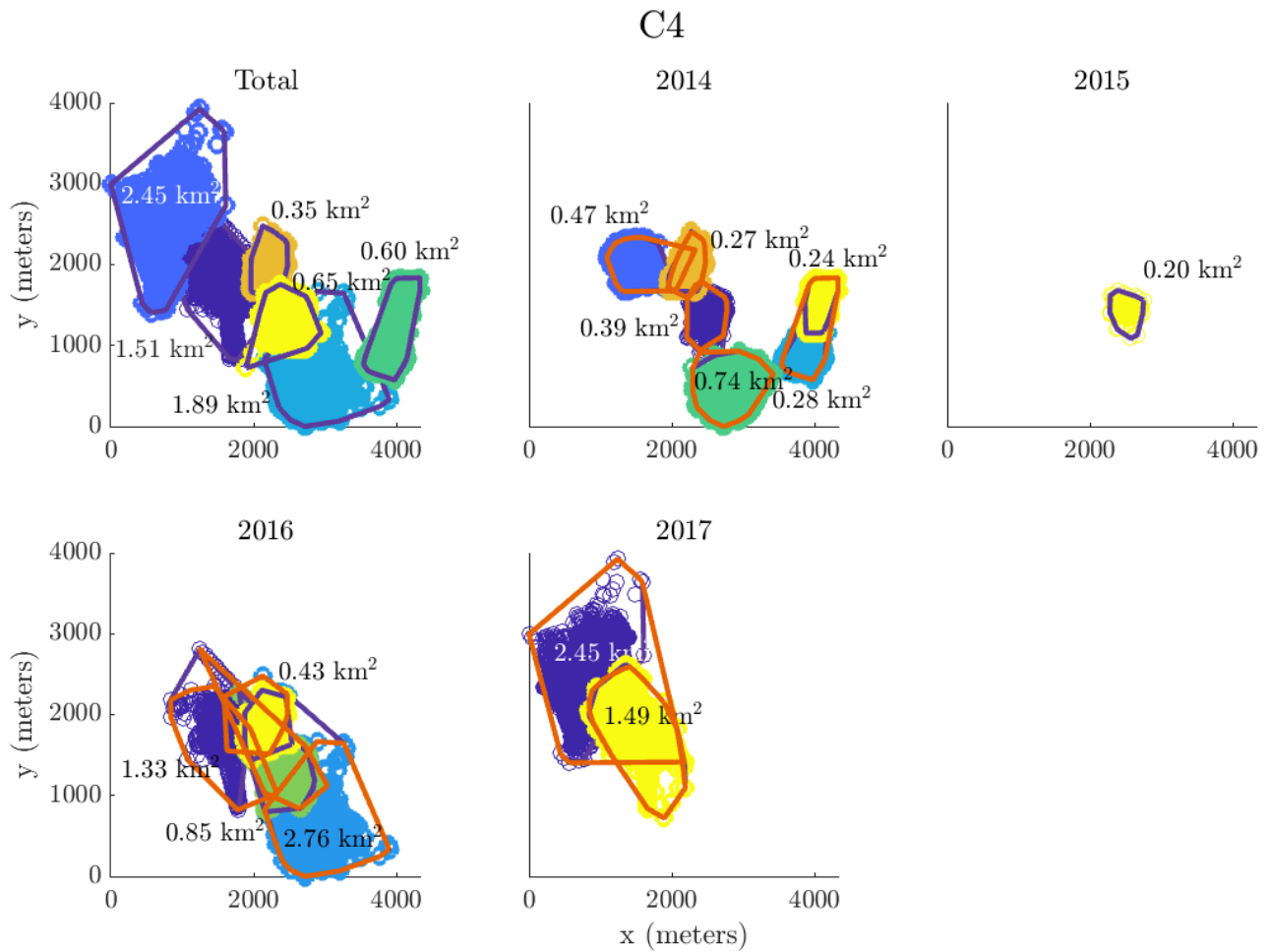

D

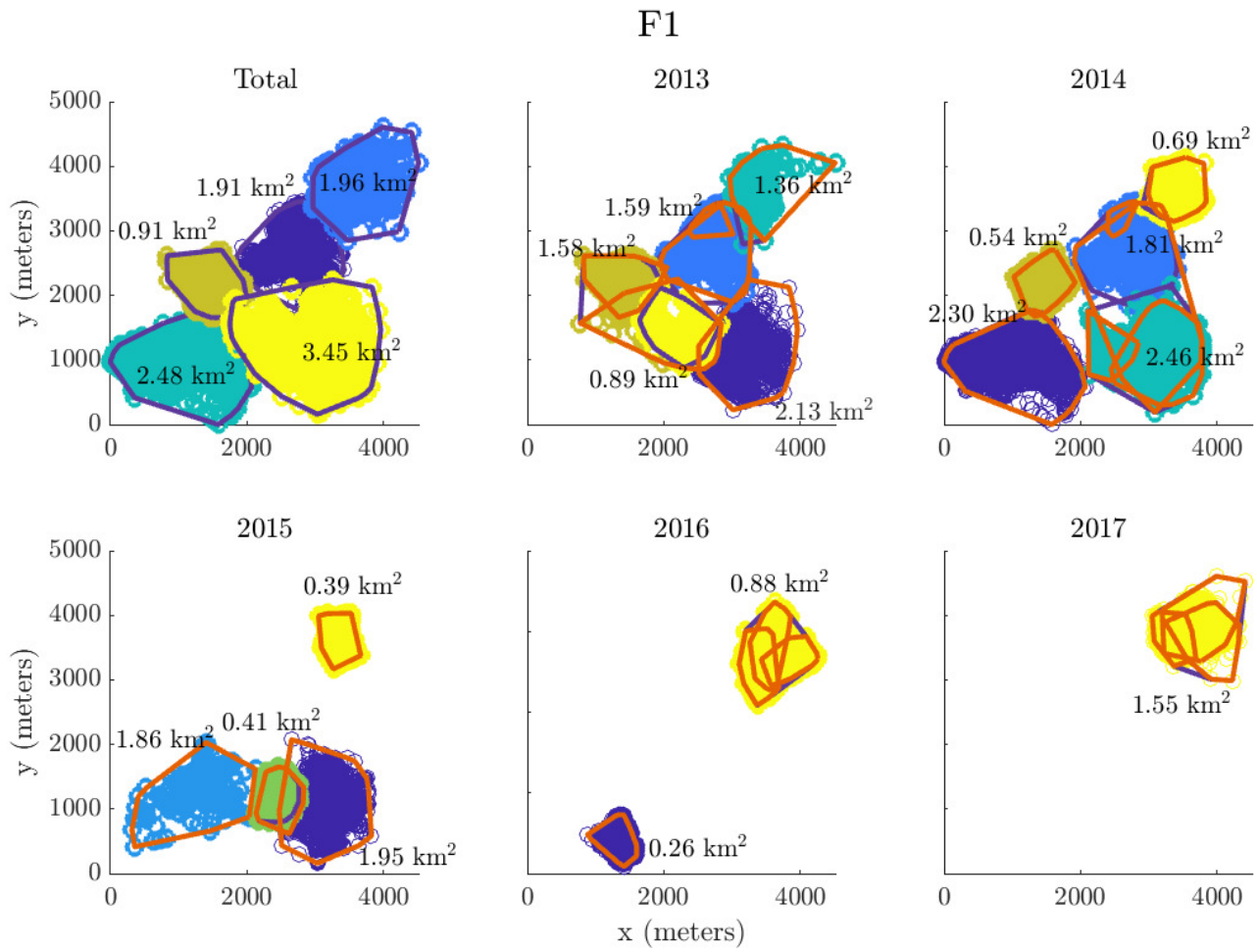

E

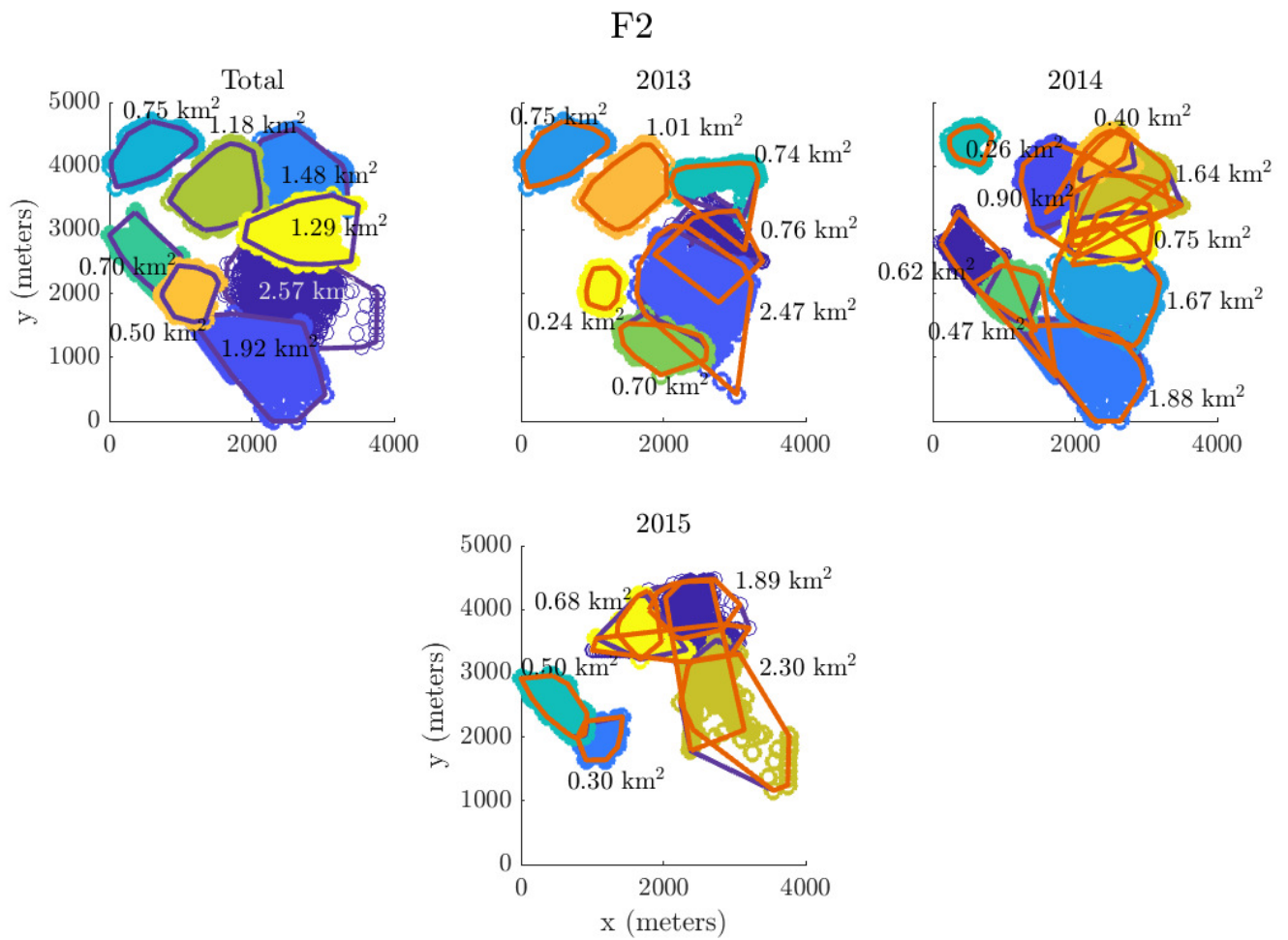

Supplement: S5 Fig — The purple lines are the convex hull of the metastable clusters. Due to the size of the Northern Ireland dataset, the areas of the clusters are not shown. Additionally, for Cornwall sites C2 and C4, there is only one social group present in 2015, so only the convex hull of the EDMD cluster is presented. (PDF) [file pcbi.1013372.s005.pdf]
