# Supplementary material for: Data-driven analysis of fine-scale badger movement in the UK
Source: PLoS Comput Biol. 2025 Aug 28;21(8):e1013372. doi: 10.1371/journal.pcbi.1013372 (PMC12393748; doi:10.1371/journal.pcbi.1013372)
Supplement: S2 Table — (PDF) [file pcbi.1013372.s007.pdf]

Table S2. Fixed effect estimates from the GLMM model L∼1+M+Sx+(1| Site:Animal).

Predictor	Estimate	Standard Error	t-Statistic	95% Confidence Interval	*p*-Value
**Intercept**	3.5866	0.032	111.3	[3.52, 3.65]	< 0.001
**Month (Ref: January)**					
February	0.105	0.024	4.41	[0.060, 0.15]	<0.001
March	0.048	0.026	1.81	[-0.0041, 0.10]	0.071
April	0.033	0.030	1.09	[-0.026, 0.092]	0.23
May	-0.008	0.033	-0.23	[-0.073, 0.058]	0.82
June	-0.119	0.039	-3.06	[-0.19,-0.042]	0.0023
July	-0.073	0.027	-2.65	[-0.13, -0.019]	0.008
August	-0.118	0.021	-5.49	[-0.16, -0.076]	<0.001
September	-0.240	0.021	-11.32	[-0.28,-0.20]	<0.001
October	-0.368	0.023	-16.33	[-0.41, -0.33]	<0.001
November	-0.442	0.024	-18.40	[-0.49, -0.40]	<0.001
December	-0.291	0.024	-12.05	[-0.34, -0.24]	<0.001
**Sex (Ref: Male)**					
Female	-0.201	0.041	-4.86	[-0.28, -0.12]	<0.001
